# Patient experiences, attitudes and expectations towards receiving information about anti-TNF medication – “It could give me two heads and I’d still try it!”

**DOI:** 10.1186/1471-2474-14-165

**Published:** 2013-05-10

**Authors:** Paul Arkell, Sarah Ryan, Ann Brownfield, Anthony Cadwgan, Jon Packham

**Affiliations:** 1 The Haywood Rheumatology Centre, High Lane, Burslem, Stoke-on-Trent ST6 7AG, UK; 2 University Hospital North Staffordshire, Newcastle Road, Stoke-on-Trent, ST4 6QG, UK

**Keywords:** Rheumatoid arthritis, Anti-TNF therapy, Health information, Patient attitudes, Counselling, HIV testing

## Abstract

**Background:**

Anti-tumour necrosis factor (anti-TNF) therapies are an important recent development in the treatment of autoimmune disease. Despite important side effects relating to immune suppression, there is lack of research into patient experiences, attitudes and expectations about the information they receive prior to starting anti-TNF therapy.

**Methods:**

In May 2011 participants were purposively sampled to form two focus groups varying in age, anti-TNF agent and pre-therapy disease activity. A semi-structured topic guide was used to explore patients’ experiences regarding the information they received prior to commencing anti-TNF therapy. The focus groups were audio-taped and transcribed verbatim. Data were analysed using content analysis.

**Results:**

Four key themes were identified.

Firstly, weighing the risks and benefits of anti-TNF therapy. However, most participants attached limited importance to side effects, saying their strong desire for RA symptom control was overriding. Two reported deliberately concealing illness in order to continue their medication.

Secondly, the desire for information. They suggested that counselling should occur at an early stage and not during a severe RA flare-up.

Thirdly, the process of starting anti-TNF. Many identified that their biggest worry was whether they would be eligible for the new medication. They remembered little about the investigations they underwent, and none said they would have objected to being tested for blood borne viruses.

Finally, the experience of being on anti-TNF. Most were positive, describing effects on quality of life as well as symptoms.

**Conclusions:**

The use of qualitative methodology in this study has enabled an understanding of patients’ attitudes towards receiving information about anti-TNF therapy. The results may be useful to health professionals in terms of the timing and content of the information given to patients prior to commencing anti-TNF therapy.

## Background

Anti-tumour necrosis factor therapies (anti-TNFs) are an important recent development in the management of many autoimmune diseases, the first of which was licensed for rheumatoid arthritis (RA) by the European Medicines Agency in 1999 [1]. With approximately 12,000 new cases of RA per year in the UK, the number of people going on to receive anti-TNF is increasing [2,3]. These medications have potentially serious adverse effects which largely relate to immune system suppression. For example, those on anti-TNF are at significantly higher risk of serious infection, and there is a theoretical increase in the risk of cancer [4].

Potential anti-TNF side effects can influence individuals’ eligibility or desire to start treatment. The importance of effective patient education regarding RA and the potential benefits and risks of treatment is therefore acknowledged as important by many authors, and is a recognised way of improving patient concordance and adherance [5]. The British Society of Rheumatology (BSR) emphasise the importance of ongoing patient education, empowerment and self-management in RA [6,7], and NICE highlight the importance of informed decision making in RA [8].

While there has been much work relating to patient preferences towards receiving general information and their involvement in clinical decision making, there is little that relates directly to anti-TNF therapy [9-12]. We therefore aimed to gain understanding of patient experiences of starting anti-TNF medication, and explore their attitudes and expectations towards the information they received during this process.

## Methods

The study was carried out in the Haywood Rheumatology Centre (HRC) in North Staffordshire UK, which serves a population of over 500,000 patients. At the time of recruitment over 2500 patients with RA were under the care of a team of 10 consultant rheumatologists and 3 nurse consultants. Two hundred and fifty of these patients were taking anti-TNF. In this unit, pre-therapy counselling for anti-TNF is undertaken by a specialist nurse who explains the purpose of treatment, how it works, and the important side-effects. Patients are asked screening questions about present and past cancer, and are screened for tuberculosis (Tuberculin sensitivity skin test and chest XRay) and Hepatitis B (blood test). An Arthritis Research UK leaflet is provided.

Twenty participants were purposively sampled from the existing list of RA patients at the hospital to give a spectrum of patient age, disease duration, pre anti-TNF disease activity and anti-TNF agent received. They were approached during routine clinic appointments and received an invitation letter accompanied by patient information leaflet which explained the study aims and methods. Ethical approval was obtained from North Staffordshire and South Staffordshire Local Research Ethics Committees. Written consent was obtained from all participants according to the Declaration of Helsinki.

In May 2011 two facilitated focus groups (one male and one female) took place in a conference room at HRC. These lasted 108 and 94 minutes duration respectively.. Gender specific groups were chosen in order to reduce any potential embarrassment whilst patients discussed topics such as sexually acquired infections (i.e. HIV) and gender specific cancers (i.e. prostate) and associated cancer screening. An introductory talk was delivered by the focus group facilitator (PA), which emphasised anonymity and confidentiality. Participants were asked open questions about their personal experience of starting anti-TNF and the information they received during this process, and whether they thought any aspect of patient care could have been improved. A topic guide (see Table 1) was developed from a literature review and discussion with a patient partner and health professionals, however participants were encouraged to speak freely and openly about their thoughts and feelings. The topics of cancer screening and HIV testing were included as it was felt that data in this area were particularly lacking. The question “how would you feel if you needed to be tested for HIV” was deliberately asked in both focus groups.

**Table 1:** Focus group topic guide


1	How did you feel about starting anti-TNF?
2	What information do you remember being given before starting anti-TNF?
3	How did you feel about the information you were given?
4	Did you do any of your own research around the time of starting the medication?
5	Was there any additional information that you would have liked to receive?
6	Were you aware of any additional tests/investigations that were carried out before starting anti-TNF?
7	When would you contact the nurse about your treatment?
8	What is your understanding about having vaccinations whilst on anti-TNF?
9	Were you given any information about any potential risks of commencing anti-TNF?
10	How did you feel about the potential risks associated with anti-TNF?
11	Were you given information about the reasons why you might have to stop your anti-TNF?
12	Are you aware of the various cancer screening programs in the UK?
13	Are you aware of the need to engage in cancer screening programs?
14	As we learn more about anti-TNF therapy it seems it might be sensible to screen patients for certain viruses such as hepatitis and HIV. How would you feel if you needed to be tested for these viruses?

The discussions were audio-taped, and written notes were taken to collect non-verbal cues. Recordings were transcribed verbatim and independently verified. Pseudonyms were used and other identifying information was removed.

Data analysis used content analysis combined with Colazzi’s procedural steps [13]. Whereas content analysis involved identifying recurring words and themes from the focus group data. The addition of Colazzi’s procedural steps provided a framework which enabled the researchers to move beyond the stage of solely identifying the themes to exploring the meaning (and its significance) relating to the themes. This approach increased the researchers understanding of the experience and information needs of patients taking anti-TNF therapy. Analysis was undertakenby two members of the study team. Each identified areas of concordance and discordance in the data, and through discussion, a set of key themes were then identified. To validate the analysis [13], written summaries of focus groups and the themes identified were provided to all participants, who were given the opportunity to respond with comments. None wished to add further comment to the findings.

## Results

Out of the 10 men and 10 women who were invited, five of each agreed to take part. Those that declined to participate were unable to attend focus groups on pre-determined dates. Characteristics of the participants are shown in Table 2.

**Table 2:** Participant characteristics

**Pseudonym**	**Age (years)**	**Time since RA diagnosis (years)**	**Current anti-TNF agent**	**Time since commenced anti-TNF (years)**	**Pre-therapy DAS28 score**
**Male group**:					
Robert	85	21	Etanercept	4	8.47
Steve	47	17	Etanercept	9	5.75
Jim	57	18	Etanercept	1	6.20
Bob	79	21	Adalimumab	7	5.57
Terry	46	11	Adalimumab	2	6.90
**Female group:**					
Katherine	64	5	Adalimumab	2	7.08
Helen	62	10	Adalimumab	8	6.07
Val	64	26	Adalimumab	7	7.94
Claire	63	15	Etanercept	6	5.92
Sally	67	21	Etanercept	4	6.53

The following four themes were identified:

1) The risks and benefits of taking anti-TNF

2) Desires for information about anti-TNF

3) The process of starting anti-TNF

4) The experience of being on anti-TNF

1) The risks and benefits of taking anti-TNF

Prior to treatment initiation, participants recalled being excited about the prospect of taking anti-TNF. Nevertheless, some said they had apprehensions about starting a new medication, and this often related to problems they had experienced with previous treatments. Perceived increased risk of cancer was also commonly identified as a concern.

“Oh yes I was worried about cancer. I mean I still am really.” – Sally

“I thought ‘increased risk of tumours’, and it really crossed my mind and I gave it a lot of thought.” – Val

“I think it’s the one that sticks in your mind [cancer].” – Helen

However, when asked about cancer screening in the UK, nobody identified this as particularly important for themselves because they take anti-TNF. Some admitted not attending screening appointments. Although there was concern about infection risk, this was not perceived as important as cancer risk:

“ Infections… oh no because I think you normally fight those off. You know as long as you are basically healthy I just presume that you can fight those off. But cancer of course is a different thing.” - Sally

Many recalled that becoming ill with an infection meant they’d had to stop taking anti-TNF. They were often more worried about this than the infection itself:

“I just don’t want to not take the drug. I put up with it and hope it goes.” – Steve

“I’ve just had a chest infection…I don’t know if it’s the anti-TNF but I’ve no intention of stopping it.” – Bob

“That was my dread, coming off it.” – Helen

They felt this way about other side effects as well, including cancer:

“I wasn’t worried about having the cancer because I thought this is something else that’s come along that I have to deal with, but in the back of my mind I thought ‘at my age now, if I’d got to come off [anti-TNF] for 5 years, I’d never go back onto it’. That really worried me.” – Val

Two women admitted trying to conceal infections in order to continue receiving their medication:

“I was trying not to sneeze or sniff or anything, or cough. Definitely don’t cough!” – Katharine

“Yes, definitely don’t cough! [in agreement with Katherine]” – Helen

Despite acknowledging the importance of side effects, most participants described the severity of their rheumatoid disease when making the decision about anti-TNF. Many explained that the prospect of symptom control was more important than any fears about drug side effects.

“When you’re that ill, if somebody told me to eat mud or clay and it might help [I would].” – Steve

“You’re scared of rheumatoid arthritis. That’s the thing you’re scared of the most… you’re scared of the pain, what it does to your joints, you know, you’re already scared. So information can’t scare you… If I’m going to get cancer, I’m going to get cancer. It can’t be any worse than rheumatoid arthritis.” - Tom

“It could give me two heads and I’d still try it.” – Helen

Many participants expressed their trust in the rheumatology team, and explained that this was why they didn’t worry about side effects.

“[Named rheumatology nurse and doctor], they’re like God to me. What they say goes! You know, I feel, I’ve got a lot of confidence in them. I feel like I trust them with my life.” - Val

“What I like is that she’s at the end of the phone … you know, if we were worried we could always get hold of her.” - Helen

2) Desires for information about anti-TNF

Participants reported getting information about anti-TNF from many different sources. Some actively sought information by doing research, while others were more passive. With regards to the quantity of information they wanted, opinions differed widely. Unsurprisingly, the few who had researched anti-TNF extensively also said they expected to be provided with very detailed information. Some talked about the danger of being given too much information:

“I can’t take it all on board when you’re telling me something. You know, I’ll get home and think ‘oh, what did she say then?’” - Sally

“I was quite happy with the information I got…I don’t think reams off the internet would have made any difference to me.” – Claire

“I feel as though I was quite well informed, but not overly bombarded with information.” – Christopher

Participants said they were often given information about anti-TNF while they were having a severe flare-up of their arthritis. Many felt that this was not a good time to receive information, and described how the activity of their rheumatoid arthritis affected the way they interpreted information:

“I was in such a hell of a state up on the ward they could have said anything to me and it would have probably gone in one ear and out the other. Because, as I said, I was really bad.” – Peter

“If you’re given a leaflet when you’re feeling that bad you just go ‘oh yeah I’ll read that later’. I mean you don’t even watch television, don’t want to read something.” – Steve

“You just focus up on that cancer, prostate cancer or whatever, just because of the mood you’re in. So sometimes you might not be in the best place to make a rational decision.” – Christopher

“When you’re really ill … you perhaps can’t make decisions as well.” – Val

There was general consensus that it would be beneficial to provide patients with information about anti-TNF at an earlier stage:

“It might be a good idea to get people with rheumatoid arthritis, whatever state you’re in, to start to become aware of the drugs that are available as the years go by.” – Val

“I didn’t know anything about anti-TNF. I’d gone through [many] different drugs … it would have been nice to know that there was something else.” – Sally

When considering counselling for anti-TNF as a whole, most participants recognised that it would be difficult to make recommendations that suited everybody.

3) The process of starting anti-TNF

Some participants admitted that their biggest worry was becoming eligible for anti-TNF, rather than the side effects:

“I just focussed on whether I could get through the hoops to actually being given the drug. When I was told ‘you pass all the criteria’, then I started thinking ‘well maybe it could cause problems’.” – Steve

“I think I became a bit of a pest in the end, you know. That was my first question before I sat down: ‘can I go on Humira yet?’” – Katharine

Participants remembered little about the investigations they underwent before starting anti-TNF. Most were aware they had some blood tests but weren’t sure what for, and some remembered being tested for tuberculosis. When exploring views on being tested for blood borne viruses including HIV, participants were open-minded about the idea:

“Well one would assume that would have been carried out automatically! [laughs]” – Peter

“So you think people wouldn’t want to have the test? … I wouldn’t mind it.” – Steve

“I can’t see why people would object to having a test for that – it’s for their own benefit isn’t it really.” - Val

One woman explained that her views would depend how a test was offered:

“I think if it was a general one, you know, if you were tested for this, this, this and this, and HIV was one of them. If it was something that happened to everybody, nobody could say ‘I’ve been picked on’ because it would be a standard thing. It would just be something else that you were tested for.” – Katharine

4) The experience of being on anti-TNF

When considering the overall effect of being on anti-TNF, participants were generally very positive. Many described a dramatic change in their physical symptoms, and some explained how they thought this had improved their quality of life:

“Outsiders, family, doesn’t matter who it is, you can’t explain the feelings and the pain you know. So for me, it’s been a godsend.” – Tom

“Well it’s changed my life. I mean I was brought in, I couldn’t walk. I couldn’t do anything. I lead a normal life now; it’s a miracle to me.” – Helen

“I’ve been tons better. I don’t really think I’m the same person I was at the beginning… I’m very happy at the moment.” – Sally

“It’s been really good the results and I’ve had no problems as far as I can tell.” – Claire

“I’ve got a good quality of life now.”- Katherine

## Discussion

The use of qualitative methodology in this study has enabled an understanding of patients’ experiences, attitudes and expectations towards receiving information about anti-TNF medication. Apprehensions about cancer were common, and many considered themselves at ongoing risk while they were taking anti-TNF. Interestingly, however, participants did not consider engagement in national cancer screening programmes as particularly important for themselves. Participants were aware of an increased risk of infection, however they were less concerned about this than cancer-risk. Most said that they considered potential drug side effects when deciding to start anti-TNF. Many, however, emphasised a far greater importance of wanting to control their RA symptoms, and very few considered declining anti-TNF. In addition, many reported that their main fear about side effects was that those side effects would result in a discontinuation of anti-TNF. Desire for information about anti-TNF varied widely, and many participants identified the need for a balance between knowing too much and too little. Many participants stated that the best time to receive information was when their RA was not actively flaring, and that it may have been useful to be given information earlier in their treatment course. Participants had anxieties relating to the process of becoming eligible for anti-TNF, and remembered hoping they would qualify. Knowledge of tests done prior to starting was generally low, and participants were open-minded about being tested for blood borne viruses including HIV.

Many authors have described how patients weigh the potential risks and benefits of different treatments, and then make a decision [11,14]. Research involving rheumatology patients has often identified the prospect of symptom control as an important factor in a treatment decisions, and this is reflected in the present work [15,16]. In their work on patient attitudes towards anti-TNF, Marshall et al. describe how some patients were desperate to try new treatments [15], and Sanderson et al. describe a key theme of patients ‘willing to try anything’ [16].

There has been much work into patient attitudes towards information provision, and how this relates to their preferences for involvement in decision making [17,18]. In a study into decision making among women with RA, at the important phase of ‘knowledge acquisition’, patients want ‘ample information about the medication presented in a straightforward way’ [11]. This appropriate information acquisition is a way for patients to reduce their anxiety, and is supported by other research on interpretation of reassurance in rheumatology clinics [19]. It is often suggested that patients who are involved in decisions about their care are more likely to comply with medication [5-7]. However, in a study of secondary care patients, 80% of patients tend to choose a ‘collaborative’ or ‘passive’ (rather than ‘active’) role in decision making [9]. A study on living with arthritis describes a spectrum of patient preference between taking charge and not taking charge [20]. Our findings are in accordance with this theory, with many patients wanting only a limited amount of information. Passive decision making seemed also to be associated with trust in healthcare professionals. In their work into perspectives of patients on COBRA combination therapy in RA, Tuyl et al. identify the importance of ‘trust in [one’s] physician. This ‘reciprocal trust’, is an important theme in a study on the doctor patient relationship in rheumatic disease [17].

We are unaware of any previous work looking into RA patients’ preferences for the time at which they receive information regarding anti-TNF therapy. Although the most convenient and common time for this to occur in the patient pathway is when they become eligible for anti-TNF, data from this study suggest this might not be the most effective nor desirable time. At the time of starting anti-TNF patients are usually unwell, and this may have effects on their ability to receive, retain and make judgements about information. It is possible that they could benefit from gaining awareness of anti-TNF at an earlier stage.

Rather than having apprehensions about starting anti-TNF, many participants described their fear of stopping the medication, which is in accordance with findings of a previous study [16]. We are unaware of data presented elsewhere which suggest patients may actively hide illness in order to remain on anti-TNF, and this an area that would benefit from further research.

In 2004, a study identified patients’ anxieties relating to assessment for eligibility for anti-TNF [15]. The present study provides evidence that these still remain, and suggests that, for some, they are more important than drug side effects.

No previous studies have explored RA patient attitudes towards being tested for blood borne viruses. The majority of patients were not concerned by blood borne virus testing, which is in accordance with recent work in general practice and acute medical settings [21]. Participants in the present study, however, were likely to be at low risk of blood borne viruses, and therefore more work to include a patient group more likely to be ‘at risk’ may be worthwhile.

The present findings represent the experiences, attitudes and perceptions of ten participants with RA regarding information provision prior to commencing anti-TNF. While this sample size would routinely be seen as adequate for this type of qualitative research, incorporating the opinions from a wider spectrum of people may have generated more themes. All participants were under the care of a single rheumatology centre, and experience of information received at other sites may be different. All participants were successfully established on anti-TNF therapy and the experience of individuals who have discontinued the medication may be different, potentially with less willingness to trade risk of adverse reaction for potential symptomatic relief, and a desire for more information about new medications.”

As participants had been taking anti-TNF for some considerable time their experiences may be affected by recall bias. It is possible they did not accurately recall the information they received or what form it was in. Importantly there was also potential for participants to confuse information relating to anti-TNF with information from previous counselling sessions for other rheumatoid arthritis medications over the years. However, the richness of the data would suggest that the commencement of anti-TNF was a major event for the participants and they could recall their attitudes and experiences with relative ease and in-depth. Other sources of potential bias were reduced through the involvement of a patient partner to inform the development of the topic guide and the validation of the data by two researchers and the participants.

## Conclusion

The authors feel that this study provides important insight into how some patients feel about starting anti-TNF therapy and the information they receive during this process. Findings may be useful to professionals throughout the multidisciplinary team in guiding patient counselling about these new medications. Further research is needed to validate our findings and expand on themes including fears about stopping anti-TNF, the deliberate concealment of illness, preferences for timing of information, and attitudes towards testing for blood borne viruses.

## Competing interest

The authors declare that they have no competing interest.

## Authors’ contributions

PA designed and planned the study, collected data, undertook data analysis, and drafted the manuscript. SR advised on study design, analysed data and helped draft the manuscript. AB was involved in planning the study, was responsible for recruiting participants, and collected data. AC was involved in planning the study, advised on infectious diseases, and helped draft the manuscript. JP conceived the study and supervised the project. All authors read and approved the final manuscript.

## Pre-publication history

The pre-publication history for this paper can be accessed here:

http://www.biomedcentral.com/1471-2474/14/165/prepub
